# Identifying Potential Gamification Elements for A New Chatbot for Families With Neurodevelopmental Disorders: User-Centered Design Approach

**DOI:** 10.2196/31991

**Published:** 2022-08-19

**Authors:** Truong An Bui, Megan Pohl, Cory Rosenfelt, Tatiana Ogourtsova, Mahdieh Yousef, Kerri Whitlock, Annette Majnemer, David Nicholas, Carrie Demmans Epp, Osmar Zaiane, François V Bolduc

**Affiliations:** 1 Neuroscience and Mental Health Institute University of Alberta Edmonton, AB Canada; 2 Department of Pediatrics University of Alberta Edmonton, AB Canada; 3 Feil & Oberfeld Research Centre of the Jewish Rehabilitation Hospital – Centre intégré de santé et de services sociaux de Laval (CISSS Laval) Centre for Interdisciplinary Research of Greater Montreal (CRIR) Laval, QC Canada; 4 School of Physical & Occupational Therapy Faculty of Medicine and Health Sciences, Research Institute of the McGill University Health Centre McGill University Montréal, QC Canada; 5 Central and Northern Alberta Region Faculty of Social Work University of Calgary Calgary, AB Canada; 6 EdTeKLA Research Group Department of Computing Science University of Alberta Edmonton, AB Canada; 7 Department of Computing Science University of Alberta Edmonton, AB Canada

**Keywords:** gamification, chatbot, neurodevelopmental disorders, engagement, mobile health, mHealth, eHealth, focus group, interview, user-centered design, health information technologies

## Abstract

**Background:**

Chatbots have been increasingly considered for applications in the health care field. However, it remains unclear how a chatbot can assist users with complex health needs, such as parents of children with neurodevelopmental disorders (NDDs) who need ongoing support. Often, this population must deal with complex and overwhelming health information, which can make parents less likely to use a software that may be very helpful. An approach to enhance user engagement is incorporating game elements in nongame contexts, known as gamification. Gamification needs to be tailored to users; however, there has been no previous assessment of gamification use in chatbots for NDDs.

**Objective:**

We sought to examine how gamification elements are perceived and whether their implementation in chatbots will be well received among parents of children with NDDs. We have discussed some elements in detail as the initial step of the project.

**Methods:**

We performed a narrative literature review of gamification elements, specifically those used in health and education. Among the elements identified in the literature, our health and social science experts in NDDs prioritized five elements for in-depth discussion: goal setting, customization, rewards, social networking, and unlockable content. We used a qualitative approach, which included focus groups and interviews with parents of children with NDDs (N=21), to assess the acceptability of the potential implementation of these elements in an NDD-focused chatbot. Parents were asked about their opinions on the 5 elements and to rate them. Video and audio recordings were transcribed and summarized for emerging themes, using deductive and inductive thematic approaches.

**Results:**

From the responses obtained from 21 participants, we identified three main themes: parents of children with NDDs were familiar with and had positive experiences with gamification; a specific element (goal setting) was important to all parents, whereas others (customization, rewards, and unlockable content) received mixed opinions; and the social networking element received positive feedback, but concerns about information accuracy were raised.

**Conclusions:**

We showed for the first time that parents of children with NDDs support gamification use in a chatbot for NDDs. Our study illustrates the need for a user-centered design in the medical domain and provides a foundation for researchers interested in developing chatbots for populations that are medically vulnerable. Future studies exploring wide range of gamification elements with large number of potential users are needed to understand the impact of gamification elements in enhancing knowledge mobilization.

## Introduction

### Background

Neurodevelopmental disorders (NDDs) include a wide range of disorders such as autism spectrum disorder, intellectual disability, cerebral palsy, and attention-deficit/hyperactivity disorder, affecting approximately 3% to 18% of the population worldwide [1-3]. The health and well-being of families with NDDs are significantly lower than that of non-NDD groups, as children with NDDs and their parents experience complex medical, social, and educational challenges [4]. Parents of children with NDDs face unique hardships, including managing communication between health care and social providers, implementing therapeutic recommendations, and maintaining their children’s medical health records while constantly advocating for their best care [5-7]. The costs associated with the care of a child with NDDs are also substantial, which results in high rates of depression and anxiety symptoms in parents [8-10]. A significant challenge that parents often experience is navigating complex health information in a short period to care of their children. However, knowledge mobilization in NDDs is not achieved easily [11-13]. Thus, the advent of innovative technological tools that facilitate knowledge sharing, such as chatbots, can significantly benefit these families [14-17].

Chatbots are artificial intelligence–based tools with natural language processing capabilities that act as web-based conversational agents mimicking human interactions [18]. Although they are not yet used in NDDs, chatbots can provide much-needed support to parents. Chatbots can conduct health surveys; generate health-related reminders; communicate with clinical teams; schedule appointments; retrieve and analyze health data; and translate behavioral indicators such as physical activity, sleep, and nutrition into diagnostic patterns [19]. Chatbots also present several advantages in the health domain in general. They compensate for staff shortages; provide anonymity, convenience, and faster access to information; and lessen the reluctance to share sensitive (eg, emotional and factual) information [20]. For instance, chatbots used for sexual health and mental health settings showed that participants were more likely to disclose information needed for treatment with a bot rather than with a human [21]. Chatbots can be positioned in a web-based environment that is well known to families, such as social media messaging platforms (eg, WhatsApp and Facebook), making them more visible to most families living with NDDs [22,23]. Thus, health chatbots are generally seen positively by internet users [24], as they can increase access to health care and improve physician-patient and clinic-patient communication [25,26].

A critical consideration when working with families living with NDDs is that it is important to engage in a sustained relationship (similar to coaching) with them to provide the best care. Their children will present different needs over time as their development emerges; therefore, we pondered whether implementing gamification can provide more sustained use of the chatbot, thus providing better care [27-29].

Gamification implements game-based mechanics such as social networks, customization, points, badges, and progress bars in nongame contexts [30-32]. Gamification has been used widely in web applications and mobile apps and assessed across various settings, including education [33] and health care [34], to increase user engagement [30,34-36]. In the health research community, gamification in mobile health applications has received considerable interest because of its potential to motivate behavior change [37-39]. Nevertheless, gamification elements have not yet been studied extensively in chatbots [40,41].

There are important caveats to consider when implementing gamification, because a product that uses gamification should not be assumed to increase user engagement [37,42,43]. Without careful consideration of the application context, user characteristics, and content quality, gamification can yield negative impacts in terms of behavior change [38,44].

### Objectives

Considering the existing knowledge gaps in gamification use in chatbots for the health domain and knowing that inappropriate gamification can potentially compromise the chatbot use [45], we aimed to (1) better understand if gamification will be considered positive for user engagement in a chatbot for NDDs knowledge mobilization and (2) discuss some commonly used gamification elements to evaluate whether they are beneficial from the perspective of parents of children with NDDs.

## Methods

### Design

Given the lack of studies on gamification in chatbots for health care, we first conducted a narrative literature review to identify potential gamification elements. We reviewed the literature from Google Scholar and PubMed, using the following keywords: “gamification,” “engagement,” “motivation,” “health care,” “education,” and “neurodevelopmental disorders” and the related diagnosis terms, “autism” and “intellectual disability.” These terms were suggested by our teams of clinical and social sciences researchers. We did not identify any gamification elements that are specific to NDDs. Nonetheless, we found several meta-analyses discussing the most common gamification elements used in web applications [35,44,46,47]. As we intended to identify elements that can be implemented in our chatbot to inform, guide, and teach parents of children with NDDs, we also examined elements that have been used more specifically in education and health care [30,32]. Then, we compiled a list of the gamification elements identified in the literature and discussed it with our research team of computer scientists and health and education professionals with extensive expertise in interacting with families of children with NDDs. This was done to prioritize the elements that can be discussed in depth with the families.

Several gamification elements were concluded to be relevant and valuable, such as *goal setting* and *social networking* [48,49] (which have been identified in clinical coaching programs and applications), *rewards* and *customization* [50], and *unlockable content* [23,24]. These elements are listed in Table 1. *Goal setting* refers to the users’ ability to create specific goals for their children (eg, behavioral goals such as potty training and bicycle riding) to help make appropriate learning and training plans. *Social networking* refers to integrating a web-based space or forum for users to discuss and share their experiences. *Rewards* indicate intangible prizes being given to the users by the application every time they reach a goal or complete a task (eg, badge). *Customization* refers to the users’ ability to change the theme of the application interface, profile picture, notification frequency, and user avatar. *Unlockable content* suggests that certain content can be restricted to users until they reach a certain level of participation or use to encourage engagement. For all the elements, visual examples were also prepared to be shown to participants, ensuring better understanding of them.

**Table 1:** Gamification elements investigated in the study.

Gamification elements	Definition	Examples
Customization	Ability to change the features of the app	Notification, avatar, and theme
Rewards	Intangible prizes for every task completed	Badges and coupons
Goal setting	Users’ ability to create specific goals	Potty training and bicycle riding
Unlockable content	Restricting contents to users who reached certain levels of participation or use	Meditation and self-help articles
Social network	Integrating a web-based space to discuss and share experiences	Forum

For a product to succeed, users’ needs must be considered during the development and implementation of that product, and later evaluation must be performed to ensure that these needs are met. User participation is essential to reflect on the values, drivers, and goals of the chatbots that are to be developed. This is evident in previous studies that highlighted the importance of user participation when developing eHealth technologies, as seen in the Center for eHealth Research road map [51]. Thus, we considered different approaches to seeking feedback from families of children with NDDs regarding implementation of gamification elements in a chatbot for NDDs. We integrated two key product design approaches: user-centered design (UCD) and double diamond approach.

The UCD approach [52] consists of several methods that take end users’ needs into account, one of which is asking end users about the tasks and goals of the application. This approach allows users to influence how an app is developed and increases users’ acceptance. UCD can reduce the development time as usability problems are identified and resolved through frequent communication with users before the system is launched [53-55].

Similarly, according to the double diamond method, there are four design steps, with the first two steps involving discovering and defining the problem before a product is developed and delivered [56,57]. Identifying which gamification features parents will find beneficial or deterring can ensure better reception of the application later.

We adapted the structures of the surveys used previously to evaluate user engagement in the postgamification application [58-60] and developed our guide for semistructured interviews and focus groups. The guide aimed to explore participants’ (parents of children with NDDs) previous experiences with technology and gamification, their opinions on the gamification elements being investigated, and their views on how they should be implemented (Textbox 1). We did not conduct a usability test as part of this project. Our goal was to identify the gamification elements that will be included in our chatbot prototype later. We will conduct usability tests in the future to test their impact on user experience.

The study was advertised with NDD-focused parent organizations, including Kids Brain Health Network, Canadian Autism Spectrum Disorder Alliance, and CanChild, via social media (Facebook and Twitter). Participants were recruited via convenience sampling [61].

**Questions and prompts that were used in the parent focus groups and interviews.**

**Main question 1**

- Can you share with the group an experience you have had with an application that uses engagement mechanisms? If you have not had one, can you please outline what you have observed from other family or friends that have?
- Prompting questions
  - What did you like about your experience?
  - What did you dislike about your experience?

**Main question 2**

- When you think of *goal setting* to improve behaviors, what are the first thoughts that come to mind?
- Prompting questions
  - How do you feel about setting your own goals in an application, as opposed to an application setting a goal for you?
  - How would you feel about *customizing* the application to send you reminder notifications about your goal for the day? (If participants like the idea of reminder notifications, “how often would you like to receive these notifications?”)
  - Would it be rewarding to receive some form of *web-based rewards* for making improvements through goal setting (eg, a web-based badge)?

**Main question 3**

- What are your thoughts on including *unlockable content* in the Chatbot?
- Prompting questions
  - What kind of surprise content would you like to see, and how could this be done well or poorly (eg, color theme)?
  - Do you think you would find this engaging? Do you think unlockable content should be included in the Chatbot?

**Main question 4**

- What comes to mind when you think about the option of including a *social network* in the Chatbot?
- Prompting questions
  - What kinds of conversations would you want to have with other parents?
  - Let’s say we include a frequently asked questions board and a regular question board where parents could post their questions. How would you feel about parents policing the quality of the responses posted by other parents? 
  - Would you use the questions section of the Chatbot if there was the potential for it to include fake news or information that was not validated by experts?
  - If we move forward with this, how would you like to *customize* the way you are represented (eg, logo, name, picture, and how much information would you like to reveal)?
  - Would you prefer for the Chatbot social network to be linked to another social networking platform such as Facebook or be independent?

**Main question 5**

- When we write up our report, what is one important point you think we should pay attention to from our discussion today?
- Prompting questions
  - What topics would you like us to talk about in the future?

### Ethics Approval

This project was approved by the research ethics board at the University of Alberta (study ID Pro00081113). All participants provided written informed consent before the sessions.

### Participants

We conducted 7 focus group sessions and 4 semistructured interviews, including a total of 21 participants, all of whom were parents of children with NDDs. Although our goal was to follow the same session format for all participants, we followed a pragmatic approach to the session type to accommodate participants who had limited time availability and flexibility or, in rare cases, preferred to be interviewed alone. In all cases (21/21, 100%), we followed the same questions, as shown in Textbox 1.

### Procedure

Owing to the COVID-19 pandemic, all sessions were conducted via the web using Zoom (Zoom Video Communications) and recorded for both video and audio. Recordings were saved on our secure server (MedIT; University of Alberta). The identifiable information of the participants was stored in a secure encrypted manner. Similarly, the audio and video recordings of the interviews and focus groups were stored in our secure server and were available only to the research coordinators and assistants directly involved in data analysis. The participants agreed to have their data collected and stored as per our written consent form. The sessions ranged from 45 to 60 minutes and used a semistructured format, containing 5 main questions and 2 to 5 prompting questions (Textbox 1).

The focus group size ranged from 2 to 4 participants, whereas interviews had 1 participant each. Owing to time constraints, and because it is commonly not feasible to focus solely on individual views in a focus group, not all participants responded to every question in the focus groups, as seen in previous studies [62,63], but we ensured to cover all questions. We also analyzed the transcripts for common responses to each question. Then, we organized the results to represent 3 main themes, capturing the consensus among participants.

### Data Analysis

Videos were transcribed using Otter.ai, a tool that uses artificial intelligence to transcribe audio. Then, the transcripts were edited manually to correct errors, remove identifying information, and ensure that all the speakers were correctly labeled. Key answers and comments were extracted and analyzed using deductive and inductive thematic approaches [64-66]. The ratings for the different gamification elements provided by participants were noted and compiled.

Open texts from the participants’ responses are included in this paper. Participants’ novel and impactful insights regarding the proposed gamification features were recorded. Participants were asked to comment on whether a gamification element was a *must-have*, *nice to have*, or *not needed* feature. Participants were not obliged to comment on every aspect. Then, the common statements from participants were used to generate the main themes using thematic analysis [67]. We followed some of the techniques of Guba and Lincoln [68], such as analyst triangulation, to establish credibility and confirmability for the study.

## Results

### Overview

A total of 21 parents of children with NDD agreed to participate, of whom 18 (86%) were White, 18 (86%) were women, and 16 (76%) were from Alberta, Canada (Table 2).

Findings from the focus groups (7 sessions; 17/21, 81% participants) and interviews (4 sessions; 4/21, 19% participants) were reviewed, which showed similar response trends; therefore, they were combined (Table 3).

The summary of parents’ input, organized into 3 main themes, is presented in Table 4.

**Table 2:** Demographic characteristics of participants of the study (N=21).

Demographic characteristics			Participants, n (%)
**Sex**			
	Female	18 (86)	
	Male	3 (14)	
**Race or ethnicity**			
	White	18 (86)	
	Asian	3 (14)	
**Region**			
	Alberta, Canada	16 (76)	
	British Columbia, Canada	1 (5)	
	Ontario, Canada	1 (5)	
	Quebec, Canada	3 (14)	

**Table 3:** Participants’ preferences toward including different gamification elements in a chatbot for neurodevelopmental disorders.

Gamification features	Participants’ response, n (%)		
	“Must-have”	“Nice to have”	“Not needed”
Customization (n=11)	4 (36)	4 (36)	3 (27)
Rewards (n=17)	N/A^a^	11 (65)	6 (35)
Goal setting (n=19)	19 (100)	N/A	N/A
Surprise or unlockable content (n=19)	N/A	6 (32)	13 (68)
Social network (n=20)	12 (60)	5 (25)	3 (15)

^a^N/A: not applicable.

**Table 4:** Summary of parents’ input, showing key themes about gamification elements (N=21).

Main themes and related key concepts		Participants, n (%)
**1. Parents of children with NDDs^a^ were familiar with gamification**		
	Participants had previous experience with gamification elements	21 (100)
	Gamification elements are beneficial or moderately effective	12 (57)
**2. Specific gamification elements should be incorporated into a chatbot for NDDs**		
	Goal setting is an important feature for the chatbot	19 (90)
	A goal template that can be personalized is needed	15 (71)
	Reminder frequency needs to be adjustable by users	21 (100)
	Unlockable content (eg, resources) is deterring or off-putting	13 (62)
**3. The inclusion of social networking is favored and the topic of medical fact-checking is controversial**		
	Social networks increase social support for parents	15 (71)
	Social networks connect parents with similar experience	16 (76)
	Social networks help parents to share good resources	21 (100)
	Social networks should be implemented	17 (81)
	Moderators are needed for social networks	14 (67)
	Medical misinformation can be displayed on social networks	11 (52)
	Medical misinformation should be filtered on social networks	9 (43)
	Parents should have control over their representation on social networks	21 (100)

^a^NDDs: neurodevelopmental disorders.

### Parents of Children With NDDs Were Familiar With Gamification

The main goal of this study was to assess whether gamification will be perceived as being potentially useful in sustaining chatbot engagement and fostering use for better knowledge mobilization. We found that all the participants (21/21, 100%) had some experience with gamification in web applications and mobile apps, and 57% (12/21) of the participants reported from their experience that the tools were beneficial or moderately effective (Table 4).

### Specific Gamification Elements Should Be Incorporated Into a Chatbot for NDDs

Our next objective was to discuss some gamification elements that have been used previously in health or education domains and evaluated to be beneficial for populations with NDDs by our team of health and social science experts.

We found robust support for goal setting, which is one of the main tools used in clinical settings for families with NDDs [69,70]. Of 19 participants who commented on goal setting, 19 (100%) rated it (eg, for behavior management) as a *must-have* feature. Of the 21 participants, 19 (90%) noted that goal setting will be very important for the chatbot and 15 (71%) proposed the idea of having a *goal template* to choose from, which can be modified to fit their child’s unique needs. When asked why a goal template is a *must-have* feature, they mentioned that goal setting will be important for new parents and recently diagnosed children:

[P]arents that are newer...they would need that template because they wouldn't know what to do.

[H]aving some suggested goals for those people who can’t think of their own goals. Because when you’re at the end of your rope, you can’t always think cohesively, actually, because a lot of our parents are burnt out or have caregiver PTSD. So sometimes, it’s hard to think of your own goals, and you already feel like you’ve tried everything. So having some suggestions that you can customize with at least as a starting point would be beneficial.

Participants provided mixed responses regarding customization (eg, color theme and reminder frequency). All participants (21/21, 100%) wanted the ability to control the frequency of reminders being sent to them when a goal is completed. This is important because parents often feel overwhelmed and pressured, as mentioned previously. Among 11 participants who commented on customization, 4 (36%) stated that it is a *must-have*, 4 (36%) agreed that it is *nice to have*, and 3 (27%) said that it is *not needed*. We showed that although some parents have interest in customization, their main goal is to gain information for their children. Participants who answered that customization is *not needed* highlighted that reminders and notifications can bring a strong feeling of pressure:

[H]aving customizable, so you can choose the frequency [notification], whether you want once a day or once a week, ...sometimes that nudge is needed. [I]f it's repeated, and it’s not wanted, it could just be adding that pressure on that you’re not doing- you already feel like you’re failing your child. And when it’s reminding you over and over again, and you don’t want it to be then you’re feeling the weight of that failure over and over again.

The parents should have...a snooze button...because it can be annoying if the parent feels pressured.

[W]hen I started...I need...daily, but after a while, I might want to change it. So I think that having it customizable will be very important. And each parent is going to have a different type of personality. And some parents might want more than one today and some parents might not want reminders at all.

[B]ecause I’m single mother, ...sometimes I just forget to check down my goals. If some apps or application could remind me to set up my goals and the follow up, ...that would be great.

Parents who rated customization as *nice to have* stressed that they value the content more than the presentation or the option to customize the color theme and user interface:

I think that you want to engage the parents to have a very visual and very easy...format...to be user friendly. And they pay attention to that...more so than...what color they can change...a lot of us parents are hungry for information, for references, links to research.

[Y]ou’re coming from a place of nothing, a lot of the time and you’re giving more than you even have, you would either feel it would add to the parental guilt, or...it would cause you to be like, disengaged. [E]ven the most willing participant who wants to change things and really wants help, you could be very overwhelmed if it was not messaged correctly.

They just want an answer. They just need help. They’re so desperate for someone to help them and find resources and access to things and the rest of it, while nice and might draw someone’s eye in...isn't necessarily going to make or break.

[P]eople want to use this app, they’re desperate. They need the information...concisely...and offering...a variety of colors across the rainbow isn’t why someone’s drawn to that app.

Parents who said customization is a *must-have* feature commented on the need to have the app tailored to individual child, as each child has very different needs:

It needs to be customizable, because parents are different, kids are different, even if they have the same diagnosis and situations are different. When you’re trying to achieve a goal, some things will work for one kid, but not for another...It needs to be customized to the particular parent and child and situation.

[I]t needs to be customizable, if you have more than one child with the extra needs.

Similarly, responses to using rewards such as badges were mixed. Of the 17 participants who commented on rewards, 11 (65%) rated it as being *nice to have*, but 6 (35%) said they would prefer not to have them. Of the 21 participants, 11 (52%) stated that web-based badges, which are a type of reward, are not rewarding, whereas 6 (29%) indicated that they are *nice to have*. The idea of the bot providing rewards may be perceived as focusing on the user (parents) and not the children with NDDs, which seems to trigger some negative feelings:

I do it not because I want to be rewarded. My reward is to see my child doing well...when we see our children do well, that’s already a reward.

I think it would be beneficial...but I think it has to make sure like it’s really tailored to this audience of parents that are totally burned out...If it’s too glossy and not meaningful...it won’t mean anything and it won't resonate. It won’t entice anyone to use it.

It’s not...something that draws me to the app. I like the functionality of the app, not the awards. But I think that some parents, especially some younger parents might need those pats on the back.

[T]hey’re not significantly motivating for me. For me, it’s more about the personal engagement. So if I want to do what it is, whatever the activity is, then I’ll do it and irrespective of things like badges or rewards. [F]or me, the engagement tool that’s most successful is tracking, ...one where I can track my progress or my participation.

However, for some parents, this can be a beneficial mode of reinforcement:

[T]hat can be a good reinforcement. And then it can, it can show the options of the reinforcement, if you are getting 50 points, you will have this medal or this badge or if you get 100 points, you’ll be getting a silver one or a gold or platinum, that is some positive reinforcement that the more we put our energy towards it and we get more successful. So the chances of success increase.

Another frequently used gamification element in apps is unlockable content. Of the 21 participants, 13 (62%) did not support unlockable content, especially if helpful information or resources were being restricted. The main goal of parents was to identify trusted and relevant resources while working under time constraints. Thus, locked content was perceived as negative and detrimental to their journey:

If I’m working on something like this, I don’t want surprises.

[T]hose parents already are motivated. [T]he focus is not trying to how to motivate them, ...is to be able to help them even more with resources, ...and not to lock it out from where they can get access to. [T]hese parents deserve to be given as much as scientists and researchers can provide.

[I]f it was unlockable content, I’d be kind of annoyed I couldn’t have it upfront if it was something I actually needed or had a question about.

I just think that if I need the information, I want to be able to access it. So making me jump through hoops to get it feels like something you’d be doing in the system, like in the public systems, jump through to get the information. I don’t feel like that’s user-friendly.

[U]nlockable content is like putting cookies on the highest shelf in the cupboard, it feels like it’s a lack of trust and a lack of understanding where we’re really coming from. [It’s like] having...affirmation...locked.

### The Inclusion of Social Networking Is Favored and the Topic of Medical Fact-Checking Is Controversial

The topic of social networks revealed the complexity of implementing a chatbot in the medical domain. Of 20 participants, 12 (60%) identified a social network as a *must-have*. Of the 21 participants, 15 (71%) agreed that a social network should be implemented in the chatbot to increase social support, 16 (76%) agreed to connect with others experiencing similar challenges, and 21 (100%) agreed to share helpful information (eg, recommended physicians and behavioral therapies):

It could be from...experiences say toilet training...or just advice on things or suggestions...It’s like a social platform.

[Y]ou get to meet new people. You get to learn about diagnosis, similarities in families, and that you’re not alone.

[I]n this app, it might be easier to connect people in a safe space to ask questions...the big thing is like feeling like you’re not alone.

Of the 21 participants, 14 (67%) indicated that rules of participation and moderation must be established to provide a safe space for parents. Comments from parents indicated the potential for emotional discussion, and its regulation should be considered:

[O]pen format for people to put comments, it can get into a heated conversation. [A]n administrator has to administer it...if there’s any language or anything inappropriate, that has to be taken out.

[W]e’re talking about a vulnerable sector, people are desperate to try something...There has to be some sort of filter or something that parents could get.

There are some extremely controversial topics that do come up in these conversations, and that’s when we need somebody else to police it. The easiest one to come up with is vaccination. It’s a very divisive topic...It’s extremely inflammatory conversations.

[H]aving a report to admin button because with our kids, there are some great suggestions that parents give other parents but [i]t could be something very dangerous...because we’ve got...parents who are at the end of their rope.

Advice that some parents give is actually dangerous as well. And when parents are at the end of their rope, they will try absolutely anything.

Opinions differed among participants regarding whether medical misinformation or anecdotal evidence should be allowed in such a network. Some participants preferred having unfiltered information displayed (11/21, 52%), including anecdotal advice posted by other parents, controversial topics, and information that has not yet been validated by experts. These participants reasoned that they preferred seeing all controversial comments and ideas and not being limited to only verified information. In contrast, others preferred being shown only scientifically verified information (9/21, 43%):

[I]f it’s posted there it needs to be validated...so the parents can see...it’s been posted by the administrator, not by parents.

Other major concerns included confidentiality, privacy, and security:

First thoughts are privacy and confidentiality and the ability to use a nickname within it so you don’t have to use your real name or divulge my personal identifiable details.

I’d probably want a little bit more anonymity than Facebook because [in] Facebook, I’m opting into...what I have revealed about myself...maybe, like demographics, like where you live, but not...the city...I don’t think people need to necessarily, for the purpose of this, ...know exactly who you are...That’s a problem that would be nicely solved in the chatbot...You’d have much more liberty to ask questions in a safer space.

## Discussion

### Principal Findings

Our study revealed several informative points regarding the implementation of gamification elements in a chatbot that supports parents of children with NDDs. All parents (21/21, 100%) were familiar with gamification and showed overall positive attitude toward integrating it into the chatbot. This is important, as such studies had not been conducted previously, despite NDDs having affected 3% to 18% of the population worldwide [1-3]. Our findings on goal setting aligned with the findings in the literature, showing that parents preferred having a customizable *goal template* for behavior management [28]. For the first time, our study showed that parents of children with NDDs found unlockable content to be deterring. This may be owing to the parents’ long journey of constantly pursuing information, which underlines the importance of adopting a UCD approach when developing a chatbot. It will be important to evaluate whether this finding is generalizable to other health domains. Our findings on social networking showed varied responses, indicating that this is a complex topic and highlighting the necessity of closely working with end users when developing a chatbot for such a vulnerable population. Although some parents preferred being shown unfiltered information on social networks, which may contain medical misinformation, this will be challenging to implement, as it can cause detrimental consequences to other parents and the medical community. The complex questions raised about social networking highlighted the importance of including users in the designing process of health-specific chatbots.

From our literature review, we identified five gamification elements that may be important for increasing user engagement in a chatbot designed for parents of children with NDDs: *goal setting*, *customization*, *rewards*, *social networking*, and *unlockable content*. From interviews and focus groups with 21 participants, we identified 3 main themes: (1) parents of children with NDDs were familiar with and had positive experiences with gamification; (2) goal setting was considered as an essential feature for a chatbot for NDDs, whereas customization, rewards, and unlockable content received more diverse opinions; and (3) although social networking was viewed positively, it is a complex feature to implement owing to the issues pertaining to medical fact-checking.

Our use of a combination of interviews and focus groups was primarily owing to parents’ limited availability. However, this allowed us to obtain distinct information. In focus groups, we were able to elicit common opinions and attitudes to form major themes, whereas interviews provided us with detailed information and unique perspectives on the same topics [71]. In addition, consulting with parents of children with NDDs, or the intended users, provided unique insight into the reasons why some gamification elements were suitable. For instance, in the case of social network, users mentioned that they would use this feature to identify other sources of information that may not be widely accessible on the web. The participants also warned about the potential negative impacts of emotional discussions on such sites.

Similarly, user consultation revealed an important aspect of creating NDD-focused chatbots. Although highly engaging in other spheres, unlockable content was overwhelmingly rated negatively. It was evident that withholding information from users, who described themselves as “desperate for information to help their children,” will be damaging. It is unknown whether this remains true in other medical domains. To the best of our knowledge, this is the first study on gamification and NDDs and one of the first studies on gamification in chatbots [41,72].

### Limitations

Our study was conducted during the COVID-19 pandemic [73-75], which, combined with the busy and fluctuating schedules of parents of children with NDDs [76-79], limited us to include only 21 parents. In addition, the convenience sampling method may have introduced a selection bias in our study. To assess the generalizability and transferability of our findings, studies in different countries and with more variable social determinants such as sex, gender, socioeconomic status, ethnicity, race, and age will need to be conducted in the future. Considering the unique vulnerability of the population interviewed, we refrained from pressuring any participant to respond to all questions. To obtain sufficient data, we included enough participants to reach a degree of saturation for each question. On the basis of our analysis, we were able to identify important themes with adequate certainty but would like to conduct further evaluation of the features identified as desirable in future prototypes or usability testing.

### Conclusions

Knowledge mobilization remains as a challenge in the medical domain [80]. This is especially true in situations of medical complexity such as public health or NDDs [81,82]. Parents of children with NDDs experience special social, medical, and financial burdens, which make it difficult for them to remain engaged in the usual knowledge mobilization tools. Gamification has been the subject of extensive research and interest, more recently in the medical field, and has been used for health professional education and patient self-management [83-85]. Chatbots have also been suggested to be used as a mental health assessment tool in the workplace [86].

Our study identified several gamification elements that should be used in a chatbot designed for parents of children with NDDs. As all our recruited participants (21/21, 100%) were parents of children with NDDs, this sample could provide a representation of the population’s responses. Recruiting parents of children with NDDs can be challenging considering their background (eg, financial and social pressure and complex demands of raising a child with NDDs). Nevertheless, understanding their perspectives is crucial for identifying gamification elements that will best suit their needs.

For the first time, we showed that parents of children with NDDs support the use of gamification in a chatbot for NDDs. Our study illustrates the importance of adopting a UCD approach when determining the gamification elements needed to be included in a chatbot for NDDs. Some commonly used elements were perceived negatively by this specific group of users. Continuous incorporation of parents’ feedback in the chatbot development will help to create a better-received application that can have positive impacts on the lives of these families. Although many studies have been conducted on using users’ feedback to improve health-centered technology, our study is the first to assess the potential reception of gamification elements to enhance the experience of users of chatbots in the health domain and more specifically in the NDD domain. Using health chatbots in the NDD domain is a practice that is still in its infancy. We believe that our study will help researchers in the same field gain a better understanding of this novel technology’s design and applications. Future studies can include prototypes incorporating different elements of gamification, which can be correlated with their impact on usability and engagement.

Our study has two main implications: users’ perception of 5 gamification elements and potential application of such elements in a chatbot that can be used as an assistant tool for families living with NDDs. Participants indicated that chatbot has tremendous potential for educating users to increase their health literacy and improve their care for children with special needs. Their feedback and perception of the 5 elements will continuously guide us in our development of a prototype for this chatbot and conduct of interviews and focus groups in the near future. Given our special targeted population, our results also shed light on the design of health chatbots for populations with NDDs, specifically to improve user experience and increase user engagement, which can ultimately improve their quality of life tremendously.

## Abbreviations

NDD: neurodevelopmental disorder
UCD: user-centered design
